# Circular RNAs in Sepsis: Biogenesis, Function, and Clinical Significance

**DOI:** 10.3390/cells9061544

**Published:** 2020-06-25

**Authors:** Jesús Beltrán-García, Rebeca Osca-Verdegal, Elena Nacher-Sendra, Federico V. Pallardó, José Luis García-Giménez

**Affiliations:** 1 Centro de Investigación Biomédica en Red de Enfermedades Raras (CIBERER), Instituto de Salud Carlos III, 28029 Madrid, Spain; jesus.beltran@ext.uv.es (J.B.-G.); federico.v.pallardo@uv.es (F.V.P.); 2 Instituto de Investigación Sanitaria INCLIVA, 46010 Valencia, Spain; rebeca.osca@gmail.com; 3 Departamento de Fisiología, Facultad de Medicina y Odontología, Universitat de València, 46010 València, Spain; elena_nacher@hotmail.com

**Keywords:** circular RNAs (circRNAs), alternative splicing, transcription, biomarker, epigenetics, sepsis

## Abstract

Sepsis is a life-threatening condition that occurs when the body responds to an infection that damages it is own tissues. The major problem in sepsis is rapid, vital status deterioration in patients, which can progress to septic shock with multiple organ failure if not properly treated. As there are no specific treatments, early diagnosis is mandatory to reduce high mortality. Despite more than 170 different biomarkers being postulated, early sepsis diagnosis and prognosis remain a challenge for clinicians. Recent findings propose that circular RNAs (circRNAs) may play a prominent role in regulating the patients’ immune system against different pathogens, including bacteria and viruses. Mounting evidence also suggests that the misregulation of circRNAs is an early event in a wide range of diseases, including sepsis. Despite circRNA levels being altered in sepsis, the specific mechanisms controlling the dysregulation of these noncoding RNAs are not completely elucidated, although many factors are known to affect circRNA biogenesis. Therefore, there is a need to explore the molecular pathways that lead to this disorder. This review describes the role of this new class of regulatory RNAs in sepsis and the feasibility of using circRNAs as diagnostic biomarkers for sepsis, opening up new avenues for circRNA-based medicine.

## 1. Introduction

Sepsis is a syndrome caused by the altered regulation of the host’s immune response to infection, which can quickly evolve into a syndrome of multi-organ dysfunction, and finally death, if effective treatments are not immediately applied. In the Third International Consensus on Sepsis and Septic Shock (SS), it was defined as “a life-threatening condition that arises when the body’s response to infection damages the host’s own tissues.” SS is “a subset of sepsis in which particularly profound circulatory, cellular, and metabolic abnormalities are associated with a higher risk of mortality than sepsis” [1].

Sepsis is one of the most common illnesses worldwide. Its incidence was 677.5 (535.7–876.1) cases per 100,000 in the world in 2017 [2], and it is increasing at a rate of 9% per year. Approximately 2% of hospitalized patients and up to 75% of patients in intensive care units (ICU) develop sepsis, of which around 30% enter into SS [3]. Despite advances in antibiotic therapy and treatments applied in ICU, sepsis has become a global problem, and is the leading cause of death in all ICUs worldwide. Rudd et al. recently reported that there are 50 million annual cases of sepsis globally, with approximately 11 million deaths, exceeding even acute myocardial infarction [2]. Moreover, sepsis has been proposed as a complication in critical patients infected by SARS-CoV-2. Specifically, two recent articles by Huang C et al. and Yang X have shown that patients with COVID-19 admitted to ICU presented lymphopenia associated with high levels of plasma cytokines [4,5], which are common characteristics in septic patients. Therefore, it has been suggested that almost all critically ill patients suffering from COVID-19 are at risk of dying from sepsis [6].

Currently, it is a priority to find biomarkers to diagnose sepsis and to identify patients who could benefit from specific therapies to lower the high mortality rates. In addition, patients who survive sepsis develop immunosuppression, which makes them more sensitive to new infections in the short term, with increased long-term morbidity and mortality [7,8]. Methods to improve early sepsis identification provide opportunities to reduce sepsis severity and deaths, as well as morbidities in survivors and the economic burden of sepsis [9].

Circular RNAs (circRNAs) are a group of endogenous RNA, with different full-length sequences, characterized by a covalently closed-loop structure that lacks poly-adenylated tails, formed by a back-splicing event; unlike linear RNAs (i.e., mRNAs), circRNAs do not have a 5′ cap and a 3′ tail structure [10,11,12].

CircRNAs were discovered in the 1970s in viruses, thanks to electron microscopy [10]. It was initially thought that this type of RNA would have a low abundance [13]. However, thanks to high-throughput sequencing and bioinformatic analysis, it is now well-known that circRNAs are common and substantial within transcriptomes [11], where they are expressed in thousands of human genes, and in some cases, demonstrate higher expression than their cognate linear isoforms [14,15,16]. The use of specific powerful bioinformatic tools based on the split mapping of RNA-seq reads has enabled the resourceful prediction of circular RNAs from RNA-seq data [15,17,18,19,20]. Recent studies have described their peculiar properties and involvement in physiological and pathological processes [21,22,23,24], and how specific patterns of circRNAs are expressed in specific tissues and development stages [10,11,15]. Moreover, circRNAs are especially highly expressed in the human brain. They are also present in most mammalian tissues [25,26,27].

Nowadays, given their role in gene regulation, high abundance, conservation in mammalian cells, and stability, circRNAs are considered very relevant molecules, with relevant functions in a wide range of pathologies [28]. In fact, RNA-seq analysis data have demonstrated the significant amount of circRNA in eukaryotic cells and their evolutionary conservation [29], and more than 15,000 human circRNA sequences have been detected in rat and mouse genomes [25,30]. Their stability was demonstrated by Enuka et al. in HeLa cells, who revealed that circRNAs have a half-life of 18.8–23.7 h, approximately 2.5-fold longer than their linear homologs [31]. Furthermore, this type of RNA is protected from common degradation pathways, which are catalyzed by nuclear and cytoplasmic exonucleases, known to control RNA abundance in cells [32]. Other studies suggest that circRNAs can regulate gene expression, by acting as miRNA sponges, interacting with RNA binding proteins (RBPs) and translational regulators [33,34,35]. In particular, the interaction of circRNAs with RBPs is considered an important part of circRNA function, in which RBPs can serve as an essential element underlying the functions of circRNAs, including their own biogenesis, and the translation and transcriptional regulation of target genes [36]. CircRNAs can also be translated in vitro and in vivo [37].

Currently, circRNAs are considered a hot research topic, because they are associated with the onset, development, and progression of a wide range of diseases [10], including neurological and cardiovascular diseases and some cancers [38,39,40]. In fact, in 2018, Zhao Z et al. demonstrated the relation between 330 circRNAs and 48 different diseases [41]. For all these reasons, and given their properties, such as enhanced stability and a high abundance in body fluids, circRNAs have been proposed as good biomarkers of various diseases, including sepsis [42]. However, the direct role that circRNAs may play in sepsis is still unknown.

This review analyzes how these regulatory RNAs play an important role in the pathophysiology of sepsis, and we propose some circRNAs as feasible clinical biomarkers. Furthermore, circRNAs have a dual role, acting as therapeutic agents and as therapeutic targets. The multimodal functioning of circRNAs opens new avenues to improve diagnosis and prognosis and increase therapeutic strategies against sepsis, thereby reducing the high sepsis-associated mortality.

## 2. CircRNAs Biogenesis

**Figure 1:**
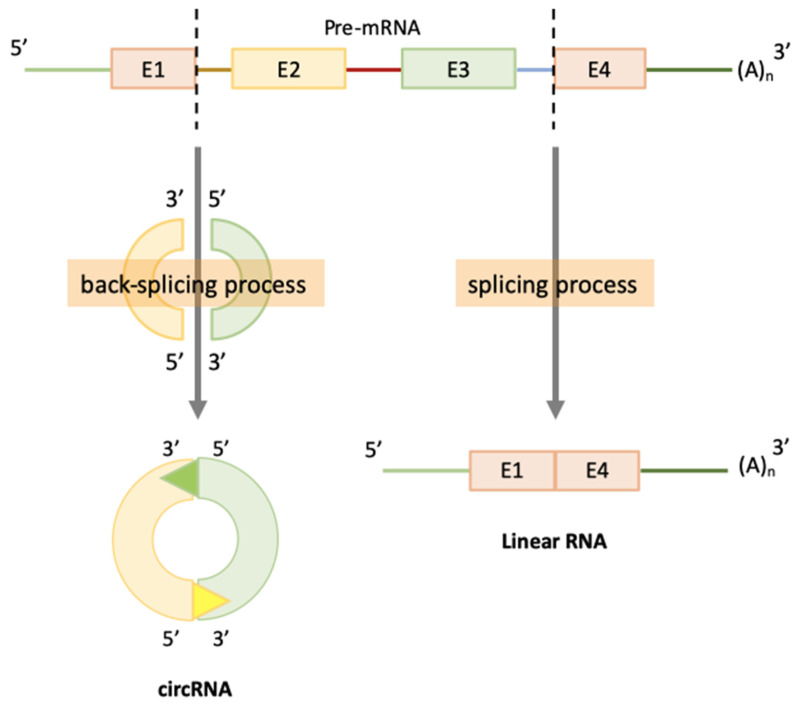
Alternative-splicing scheme for circRNA formation. (Left) The back-splicing process in which circRNAs are formed from covalently closed linear RNA. Triangles indicate the donor site located at the 5′ splice site. (Right) The canonical splicing process in which pre-mRNA gives rise to linear RNA.

CircRNAs are produced mainly by the transcription of protein-coding gene’ exons by RNA polymerase II (RNA-pol II) [22,43], but they can also contain introns [44,45]. This kind of RNA is not usually generated by the same canonical RNA splicing as linear RNA [43,46,47,48,49]. CircRNAs are produced by a pre-mRNA back-splicing process, which is mediated by a spliceosome, and is able to link an upstream acceptor splice site (3′ splice site) to a downstream splice donor site (5′ splice site) on the same exon or others [12,15,29,43,49,50,51] (Figure 1). CircRNAs biogenesis is typically done at the expense of canonical mRNA isoforms, which suggests that circRNAs compete with the maturation of their linear counterparts. So, circRNAs appear to be important regulators of mRNA production [29,43,51]. In addition, a single gene locus is able to generate many circRNAs through alternative back-splice site selection, compared to canonical RNA splicing [18]. This fact contributes to producing lower concentrations of linear RNAs with the original function (“parental linear RNA”). Therefore, due to the back-splicing process, a different molecule of linear RNA forms with a changing function that has a substantial biological impact [20,52,53]. Jeck et al. have shown this to be the case in human fibroblasts, which express 10-fold more circRNAs than their linear RNA counterparts [14].

**Figure 2:**
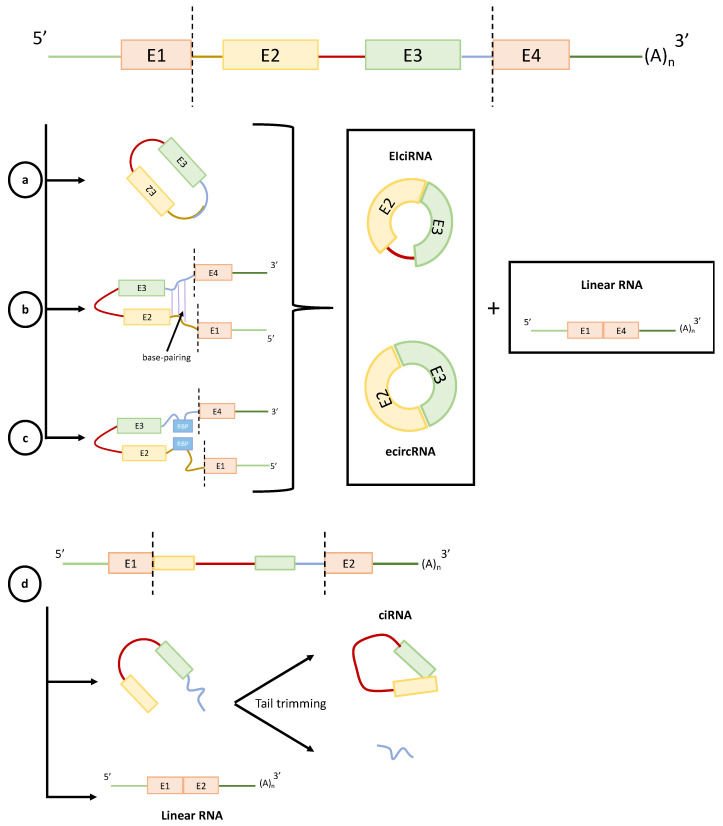
RNA circulation biogenesis processes all produce a linear molecule and different circRNA structures. (**a**) Lariat-driven circularization. This process results in ecircRNA or EIciRNA development; (**b**) Intron pairing-driven circularization. This occurs with the making of an intronic base pairing composed of complementary sequences (i.e., Alu elements). The intron-pairing process is followed by back-splicing and exon circularization; (**c**) RNA binding proteins (RBPs)-driven circularization. Proteins able to join the two intronic flanking sequences to facilitate the RNA circularization process; (**d**) Circular intronic RNA.

There are three classes of circRNAs, depending on the pre-mRNA material from which circRNAs are made: (1) exonic circRNA, which derives from back-spliced exons (ecircRNA); (2) circular intronic RNA (ciRNA), which originates from spliced introns; and (3) exon-intron circRNA (EIciRNA), which come from circRNA containing both exons and introns [54]. It has been reported that ecircRNAs are exported to the cytoplasm (where they are most often detected), while some ciRNA and EIciRNA are retained in the nucleus [47,55,56]. Jeck et al. proposed that circRNAs can be produced through two different pathways. The first is called lariat-driven circularization (Figure 2a), and is associated with an “exon skipping” process, which consists of a covalent splice from the 5′ end site (donor) to the 3′ end site (acceptor) [14,15,29,43,49,50,57]. Afterwards, the lariat is joined to the spliceosome by removing introns and forming an exonic circle [10]. The second pathway proposed by Jeck et al. is known as intron-pairing driven circularization (Figure 2b), and is based on pairing complementary motifs in transcripts. Alu elements are suggested as important regulators of circRNA biogenesis, but other inversed repetitive sequences are also adequate to drive RNA circularization [10,14,49]. Some studies indicate that the intron-pairing-driven model might occur more frequently than lariat-driven circularization [44]. Another process exists and is able to form circRNAs by joining RNA molecules flanking introns through RBPs. Among these proteins are, for example, Quaking protein (QKI) and Muscleblind protein (MBL) [28,43] (Figure 2c). Thus, the QKI protein is responsible for regulating circRNA abundance in human cells, through the binding of the 3′ and 5′ ends of circularized exons, and is also responsible for facilitating dimerization and mediating their splicing [28]. Other studies in human cells have shown that circRNAs can be formed by intron flanking joining mechanisms because of debranching failure [58] (Figure 2d). It has been demonstrated that during sepsis, there is an alteration in the alternative RNA splicing patterns, which results in the disturbance of the patient’s immune response, mainly due to the attenuation of B and T lymphocytes [59], contributing to critical phenotypes. Although it is not very clear how the alteration in the alternative splicing process occurs during sepsis, it is known that alternative splicing is a key process in the generation of circRNAs. Moreover, it has been shown that small changes in the process critically affect their biogenesis, affecting the concentration of some circRNAs by modulating their expression [60]. Furthermore, changes in alternative splicing patterns can also give rise to different circRNAs, modulating a wide range of molecular mechanisms and altering their physiological state [43,61].

Due to the role that circRNAs play in the modulation of different cytokines and immune proteins [62,63], altered states of alternative splicing in sepsis may alter the expression of circRNAs, which could partially explain the changes in the immune response of septic patients. However, this is still an unexplored field, so, although it has been attracting a lot of interest in recent years, more studies are required to demonstrate how the alteration of the alternative splicing may affect the biogenesis of circRNA during sepsis. In this regard, the fact that global transcription and translation profiles are altered during sepsis [64] lends support to the idea that changes in alternative splicing patterns may play a key role in sepsis, perhaps by modulating circRNAs biogenesis.

Interestingly, circRNAs are aberrantly expressed in many diseases and exhibit roles, such as miRNA sponges, protein decoys, transcription regulators, and regulators of translation. Despite the specific mechanisms involved in the dysregulation of circRNAs in different pathologies like sepsis, they are not completely understood, because distinct factors contribute to this dysregulation, such as their biogenesis from parental genes, export from the nucleus to the cytoplasm, and cell removal, among others. In line with this, aberrant *cis*-elements seem to be important regulatory components in circRNAs production, especially in humans [43]. Abnormal spliceosomal machinery and aberrant transactivating factors have also been postulated as central players in the biogenesis of circRNAs [28,43,65]. In this regard, despite the initial research concluding that splicing events occur co-transcriptionally in most cells and tissues [66,67], recent research demonstrates that the majority of circularizations occur post-transcriptionally [18,68], and splicing and transcription elongation are mutually dependent [69]. Furthermore, several mechanisms have been put forward to explain the role of the epigenetic aberrations involved in the dysregulation of circRNAs. Accordingly, DNA methylation, chromatin remodeling, and post-translational modifications of histones directly impact circRNAs production [18,68], for example by controlling different alternative splicing events during circRNAs biogenesis [12]. 

The biogenesis process of circRNAs inherently lowers parental RNA levels, which may lead to a reduction in mRNA and, in turn, to low levels of translated proteins [59], thereby producing the deregulation of a wide range of cellular processes. Finally, circRNAs can also be produced from intronic sequences with no clearly defined function, which may not lead to the physiological worsening associated with low specific linear RNA levels, but then again, may do so, because it is extremely uncommon, and this biogenesis type is expected to occur in a lower proportion than others.

As some of the above-described mechanisms are altered in sepsis, it is plausible to hypothesize that abnormal levels of circRNAs and low levels of linear RNAs concentrations due to the inherent biogenesis process of circRNAs may affect the abundance of the various immune mediators and transcriptional factors involved in the inflammation and immune response. We will consider this below.

## 3. Technologies Available to Analyze CircRNAs as Biomarkers

One of the main reasons for the late discovery of circRNAs lies in the extreme difficulty in finding them, mainly because it is hard to distinguish them from other small RNAs, such as miRNAs. Fortunately, RNA-seq and bioinformatics may help us to understand the pathways that produce circRNAs and how these pathways modulate the different molecular responses in a wide range of diseases [14,15,22,53].

Current detection methodologies require circRNAs to lose their circularity in order to detect them. Since circRNAs were discovered, several tools have been developed to analyze their expression, and to validate that circRNAs indeed exist [70]. In parallel to molecular strategies, bioinformatic tools have also been developed to identify new circRNAs and to quantify their expression with high fidelity.

Jeck et al. published a new protocol in Nat Biotechnol [44] called Circle-Seq, which consists of using the RNase R enzyme to process linear RNAs, while circRNAs remain intact. It has recently been shown that it is very difficult to determine the circularity of an RNA transcript by using only this treatment, because some circRNAs are sensitive to this enzyme [33], which could cause false-negatives and lead to biases. The use of other, additional methodologies to isolate circRNAs has also been proposed, such as 2D (two-dimensional) denaturing polyacrylamide gel electrophoresis or ribosomal RNA (rRNA) depletion and poly(A) depletion, to increase the amount of circRNAs in samples for RNA-seq [53]. Nonetheless, their actual efficacy in clinical practice is unknown.

The most widely used method to validate and quantify circRNAs is reverse transcription-PCR (RT-qPCR). This method is implemented by using, for example, a strategy based on RNA treatment with and without RNase R, followed by a step of reverse transcription to cDNA. The cDNAs from RNA with or without RNase R treatment are then analyzed by PCR amplification, with primers specifically designed for each isoform by a PCR reaction to detect the presence of both circRNAs and specific circRNAs through the design of specific primers, which are not generated by the normal splicing occurring during mRNA and other small regulatory RNAs processing [71]. The great advantage of this method is that it may be widely used in clinical practice, and its implementation as a diagnostic biomarker detection tool is simple and cheap.

The use of a microarray as a diagnostic method is also possible, thanks to its sensitivity and specificity. However, there is some concern about this approach, as only the circRNAs included in the array can be evaluated, which means that newly discovered circRNAs cannot be included with microarray technology. Thus, it is extremely difficult to determine the absolute amount of circRNAs. Hence, microarrays are a good approach for determining the relative expression levels of circRNAs in comparisons made of different exploratory groups.

RNA-sequencing (RNA-Seq), coupled with directed bioinformatic analysis, has notably contributed to discovering and characterizing circRNAs. In fact, RNA-Seq has yielded many circRNAs and contributed to the discovery of the intrinsic characteristics of circRNAs [14,29,44,72,73]. More importantly, disease-relevant circRNAs can be detected in human peripheral whole blood by RNA-Seq [74,75].

In order to overcome the challenge of discovering new circRNAs that contribute to comprehend and diagnose sepsis, a number of bioinformatic and statistical methods have been described. Moreover, many tools have been designed to decipher whether resulting circRNAs are exonic, intergenic, intronic, or UTR [15,29,76]. The results have been deposited in specialized databases like circBase [77] and CIRCpedia [18].

Some currently used bioinformatic tools are designed to process RNA-seq data and to identify circRNAs [78,79]. However, despite the central role that different predictive software and algorithms can play in discovering circRNAs, their implementation in clinical practice is still a distant solution. However, their vast potential is undeniable, and they are expected to be implemented in clinical routine in the coming years, with the support of the latest technological advances. Moreover, several tools and databases are appearing which contribute to the understanding of the different functions of circRNAs and the role they can play in different diseases [80]. These tools help to identify the circRNAs that competitively sequester miRNAs, by preventing them from interacting with their natural targets. This process is highly relevant in sepsis, because miRNAs are key regulators in inflammation, endothelial dysfunction, and immunosuppression during sepsis [81,82].

Furthermore, there is an evident need to understand the role that circRNAs play in sepsis. Future research is required to uncover not only the molecular role of different circRNAs related directly and indirectly to sepsis, but also the new circRNAs that modulate the great heterogeneity of sepsis, which can then be used as biomarkers of diagnosis, prognosis, and/or theragnosis.

## 4. CircRNAs Function in Sepsis

**Figure 3:**
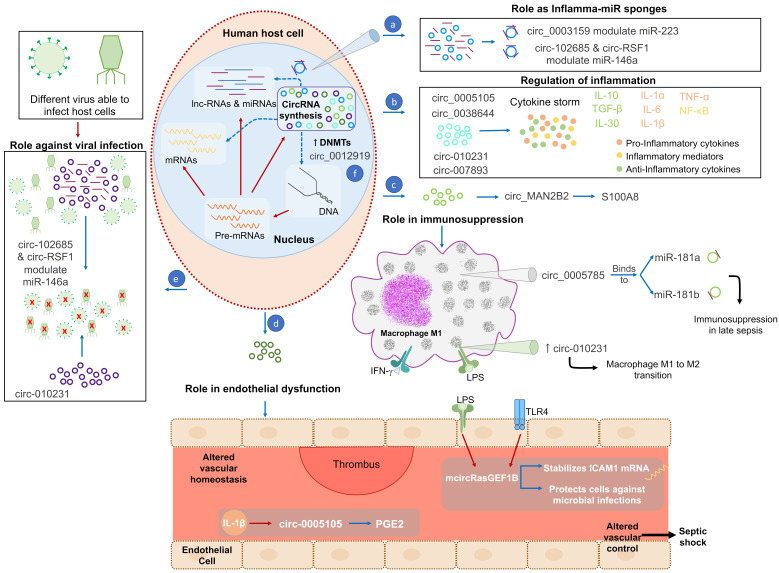
CircRNAs controlling several molecular mechanisms in sepsis. During sepsis, different molecules induce the activation of circRNAs biogenesis. (**a**) CircRNAs are able to bind to miRNAs and modulate their expression by inhibiting their functioning. (**b**) CircRNAs regulate inflammation by controlling the expression of inflammatory mediators and pro-inflammatory and anti-inflammatory cytokines. (**c**) Immunosuppression is also mediated by circRNAs, for example by mediating the control of immunosuppression mediators such as S100A8, or of macrophages or other immune cells. Notably, when macrophages bind LPS or INFγ, they produce circRNAs, which can act directly or through miRNA binding. (**d**) CircRNAs during sepsis participate in endothelial dysfunction and alter vascular homeostasis by producing a thrombus. (**e**) Specific circRNAs compete with viral mRNA to help host defense. (**f**) Finally, circRNAs have the capacity to increase DNMTs production, thereby altering transcription in immune cells. LPS: lipopolysaccharide; INFγ: interferon gamma; TLRs: toll-like receptors; mRNA: messenger RNA; lnc-RNA: long non-coding RNA; miRNA: microRNA; CircRNAs: circular RNAs; DNMTs: DNA methyl-transferases. Blue arrows indicate the processes activated by circRNA and red arrows denote signaling or activated processes. The red “x” inside viruses indicates virus neutralization.

Very little information is available about the role of circRNAs in sepsis, because the elucidation of the role that these molecules play in human diseases has become relevant only in recent years. However, circRNAs may play a key role in sepsis because of their ability to modulate different molecular mechanisms [10], including inflammation [83] and immune response [62], and to control multiple biological processes in metabolic organs (i.e., liver, pancreas [84]) (Figure 3 and Table 1). Moreover, the identification of the mechanism by which host circRNAs can bind viral mRNAs merits special attention, because it indicates that circRNAs are likely to resist viral infection [85] (Figure 3). Therefore, circRNAs may play a key role in host defense against viruses.

### 4.1. Role of CircRNAs in Inflammation

It is known that one of the first molecular responses in sepsis is the “cytokine storm”. During sepsis, the “cytokine storm” mediates the initial pro-inflammatory phase by releasing proinflammatory cytokines, such as IL-1α, IL-1β, tumor necrosis factor-α (TNF-α), IL-6, and interferon gamma (IFN-γ), among others [86]. Nevertheless, during a septic process, anti-inflammatory cytokines (i.e., IL-10, IL-30, transforming growth factor-β (TGF-β), etc.) are also produced and continuously released, strongly influencing the sepsis progression and outcome [87].

The importance of miRNAs in controlling sepsis pathophysiology has been demonstrated. In fact, both host miRNAs and DNA virus-encoded miRNAs are involved in the sepsis-induced cytokine storm, leading to increased inflammation, and even to subsequent immunosuppression. In fact, it has been widely demonstrated that miRNAs are able to regulate different key cytokines expressed during sepsis and significantly mediate their expression as TNF-α [88,89,90], IL-6 [91,92], NFκB [93,94,95], IL-10 [87], IL-18 [96,97], IL-27 [98,99], and other pro-inflammatory and anti-inflammatory cytokines with differential expressions in sepsis.

Although very little information exists about circRNAs in sepsis, it may be hypothesized that the regulation exerted by circRNAs plays a fundamental role in different sepsis stages (Figure 3). In fact, circRNAs control the expression of the key proteins and cytokines that participate in sepsis (Figure 3, Table 1). For example, circ-4099 is induced by inflammatory mediators, such as TNF-α [100] and circRNA_0038644, and has been demonstrated to regulate the expression of NF-κB in sepsis [101], while IL-6 is regulated by circ_007893 [102]. Moreover, circRNA_0005105 facilitates the expression of inflammatory cytokines [103], thereby mediating a pro-inflammatory phenotype which is critical in sepsis pathophysiology. The findings of Zhang et al. support the role of circRNAs in mediating inflammation, by showing how circ_0012919 was abnormally up-regulated in CD4^+^ T cells in patients with a hyper-inflammatory syndrome, such as systemic lupus erythematosus (SLE). circ_0012919 also increases the expression of DNA methyl-transferase 1 (DNMT1), modulating the immune response by reducing the expression of CD70 and CD11 in CD4^+^ T [104].

Interestingly, circRNAs may function as “molecular sponges”, by controlling the expression of different types of non-coding RNAs, such as miRNAS, involved in regulating different processes in sepsis [105,106,107,108]. One of the best characterized miRNAs in sepsis is inflamma-miR miR-223, which participates in the regulation of innate immunity by controlling the activation and differentiation of neutrophils and macrophages [109]. The down-regulation of miR-223-3p induces the expression of IL-6, IL-1β, and TNF-α, which supports the key role played by miR-223 in innate immunity regulation [110]. Interestingly, circ_0003159 has recently been shown to be able to regulate the expression of miR-223 [111], providing a new mechanism to explain the low levels of this miRNA in sepsis patients [112].

Another inflamma-miR described in sepsis is miR-146a, which is involved in several key processes in sepsis, such as the control of innate immunity, endotoxin tolerance and immunosuppression, inflammatory response, antiviral pathways, toll-like receptors (TLRs), and cytokine signaling [113]. Importantly, miR-146a has proven its ability to predict 30-day mortality in septic patients [114]. In this scenario, two circRNAs, namely circ-102685 and circ-RSF1, modulate the expression of miR-146a [115,116]. The two circRNAs may be postulated to be modulators of inflamma-miRs, thereby suggesting their role in inflammation during sepsis.

Chen J. et al. have demonstrated that circ-PVT1 binds miR-125 family miRNAs to inhibit their function [117]. This family is especially interesting in sepsis, because miR-125b correlates with sepsis severity, inflammation, and increased mortality in septic patients [118]. In fact, circ-GLI2 is a circRNA that specifically targets miR-125b-5p [119].

miR-192-5p, miR-26a, and miR-191-5p have been postulated as key biomarkers in sepsis, for their ability to discriminate between sepsis and other severe inflammatory cases, for instance severe systemic inflammatory response syndrome (SIRS) [120].

Zhongrong Z. et al. revealed that in intervertebral disc degeneration, circ-MSR regulates the expression of miR-27 [121], which is up-regulated and promotes an inflammatory response in sepsis [122].

### 4.2. Role of CircRNAs in Immunosuppression

Different non-coding RNAs have been postulated as key mediators of sepsis, for their ability to control the innate and adaptive immune system, in addition to simultaneous pro- and anti-inflammatory phenotypes (Figure 3 and Table 1).

Besides the role of some circRNAs in regulating inflammation, other miRNAs may also regulate the immune system. For example, circ_0005075 is involved in the regulation of miR-23a-5p and miR-23b-5p, which control key events in sepsis pathophysiology. Moreover, miR-23a-5p is up-regulated in sepsis, contributing to acute respiratory distress syndrome induced by lipopolysaccharide (LPS) [123]. Furthermore, miR-23b-5p up-regulation controls T-cell apoptosis through NF-κB signaling, as demonstrated in a mouse model of sepsis [124]. Interestingly, the inhibition of miR-23b-5p causes down-regulation in programmed death ligand 1 (PD-L1) expression in splenic T-lymphocytes from septic mice, by reducing late-sepsis-induced immunosuppression and improving survival [124]. These results suggest that circ_0005075 could be used in a possible therapeutic approach in sepsis, as suggested previously for cancer therapy [125].

A recent study explored the expression of circRNAs in macrophages under two different polarization conditions: M1 macrophages induced by IFN-γ and LPS, and M2 macrophages induced by interleukin-4 (IL-4). The results showed 189 circRNAs with differential expression in the M1 compared to M2 macrophages, which have a relevant role in producing anti-inflammatory cytokines such as IL-10 and IL-13. Of the 189 identified circRNAs, circ-010231 was the most overexpressed circRNA in M1 after LPS stimulation [126], which contributed to M1 to M2 polarization of macrophages and suggests that this circRNA is a good candidate to be explored in human sepsis. Therefore, knowledge of the entire molecular process contributing to M1 to M2 transition is extremely important, because it opens new therapeutic possibilities by controlling immune responses and immunosuppression. Another circRNA, circ_0005785, can bind to miR-181a and miR-181b—two miRNAs which have been previously postulated as promoters of immunosuppression in late sepsis [127].

Finally, circ_MAN2B2 regulates S100A8, which is another important protein involved in immunosuppressive states in sepsis [128].

### 4.3. Role of CircRNAs in Endothelium Dysfunction

Impairment of the endothelial function is one of the most important physiopathological hallmarks of sepsis [129,130,131]. Endothelial function is critical for maintaining vascular homeostasis and activating different processes, such as thrombosis, inflammation, and vascular remodeling [132]. Hence, it has been shown that the outcome of sepsis substantially improves when endothelial dysfunction is avoided [133,134,135].

Regarding inflammatory phenotypes and the endothelium, Liu et al. found that the expression of circ-CER was up-regulated in chondrocyte with catabolic stimulators like IL-1 and TNF-α [136]. Moreover, Wu et al. observed how circ-0005105 stimulated the expression of nicotinamide phosphoribosyltransferase (NAMPT), and the generation of IL-6, IL-8 and prostaglandin E2, due to the down-regulation of miR-26a expression [103], while IL-1β promotes circ-0005105 expression in a chondrocyte extracellular matrix degradation model [103] (Figure 3 and Table 1).

In a recent study, Tie-Ning et al. counted 11 circRNAs with associated differential expressions in an LPS-induced rat SS model with septic myocardial depression [137], one of the main causes of death associated with sepsis when the endothelium plays a key role.

One of the most plausible biomarkers for sepsis is mcircRasGEF1B. This circRNA regulates the stability of mature ICAM1 mRNA and can protect cells against microbial infection (Figure 3). In fact, a recent study revealed that mcircRasGEF1B is induced after LPS stimulus during microbial infection [138]. The mechanisms in endothelial cells may be similar to those in LPS-stimulated mouse macrophages, in which Ng et al. observed that only mcircRasGEF1B was regulated by the TLR4 pathway [138].

## 5. Clinical Significance

Infections are very common in young and old people worldwide. In most people, the host’s immune response suffices to deal with a potential threat, but in some cases, infection may be associated with an inadequate or inappropriate host response, by mediating the development of sepsis [1,148,151,152].

As there is no specific treatment for septic patients, their management is based on attempting to control infection and supporting the different organs whose functions may be compromised [153]. In fact, one of the biggest problems is that patients deteriorate quickly, progressing to SS and multiorgan failure if not treated promptly and effectively. It is noteworthy that early treatment has been shown to improve patient outcomes [153,154,155], but early treatment depends on promptly recognizing and diagnosing sepsis, which may contribute to rapidly starting the appropriate therapy [153]. Nevertheless, the early diagnosis of septic patients remains a challenge for clinicians and researchers around the world.

Because of all the aforementioned difficulties in making a correct early sepsis diagnosis, the availability of precise biomarkers would be extremely useful to allow proper and timely treatment to start, thus maximizing the possibilities of patient survival. To date, more than 170 biomarkers have been proposed and clinically evaluated [7], including various cytokines, receptors, cell surface markers, coagulation factors, complement factors, and acute phase reactants, among many others [156,157,158], but none offer anything near 100% specificity for sepsis.

Apparently, circRNA modulates a wide range of molecular responses related with immune system control, inflammation, and endothelial function, which are relevant biological processes altered in sepsis. In this regard, the dysregulation of specific circRNAs has been related to the development and progression of sepsis [126,137,138]. A number of studies report associations between circRNAs and almost all the miRNAs are postulated as biomarkers in sepsis, most notably with the different cytokines that have an abnormal expression in sepsis, inducing the characteristic “cytokine storm” which contributes to multiorgan failure. In light of this, circ_001569 directly inhibits the transcription of miR-145 [140], thereby controlling TGFBR2 levels in lung tissues [159] and playing a direct role in the molecular signaling responsible for sepsis-induced acute lung injury.

Other circRNAs, such as circ_HIAT1, bind to miR-29a-3p and miR-29c-3p. In this case, circ-HIAT1, also known as circ_0000096, performs a “miR reservoir” function by increasing miR stability in human atherosclerosis and some cancers [141], unlike the classic function of circRNAs that act as an “miR sponge”. Interestingly, circ-HIAT1 also targets matrix metalloproteinase (MPP)-2, and MMP-9 in solid tumors [160], which are elevated in the plasma [161] and lung tissue [162] of patients with severe sepsis.

Notably, circ-MYLK and circ-CTDP1 are also able to target miR-29a-3p [163,164]. The targeting of miR-29a-3p is of the utmost importance, because high serum levels of this miRNA produced by immune cells have a good predictive value when assessing the 28-day mortality of sepsis patients (Table 1).

Circ_NSD2 is another interesting circRNA to be explored in sepsis, because it regulates different processes through the sponging of miR-199b [142], a miRNA with higher levels found in total serum and blood cells (leukocytes, erythrocytes, platelets), and previously demonstrated to possess the early ability to discriminate SS from sepsis patients [165].

Another important point to consider is that sepsis is usually associated with bacterial infections, but it can also be induced by viral and fungal infections, although the inflammatory response is generally less marked in these cases. In particular, viral sepsis lacks a definite diagnostic criterion [166]. So, circRNAs could represent a major achievement in viral sepsis diagnosis and offer several advantages over other biomarkers. For example, circRNAs remain stable in the presence of viral infections. In fact, competitive binding has been demonstrated between host circRNA and viral mRNAs, which indicates that circRNAs participate in the host defense against viral infections [85]. These findings seem to indicate that specific circRNAs are expressed in the presence of viral infections by postulating circRNA as good biomarkers to diagnose the origin of sepsis and establish a prognosis for septic patients.

Moreover, circRNAs are both tissue-specific and developmental stage-specific; their expression has been related to the initiation and progression of many disease types, such as neurological, cardiovascular, and cancer [38,39,40]. Given their structural conformation (covalently closed loop that lacks free 3′ and 5′ ends), circRNAs are very stable in blood [74] and resistant to exonucleases. Indeed, circRNAs RNAs show an average half-life of about 48 h compared to the 10 h of linear RNAs in plasma [15,31,44]. This confers upon them the capacity, not only to be feasible biomarkers, but also to act as theranostic tools, which means that circRNAs may serve as circRNA-based diagnostic and therapeutic agents in sepsis patients.

Nevertheless, sepsis pathophysiology is very complex, and understanding the molecular mechanisms that guide disease complications and fatal outcomes is still a prerequisite to finding effective biomarkers and promising treatments to reduce the high morbidities and mortality in sepsis survivors. Obviously, the identification of circRNAs opens new avenues to understanding sepsis and learning about other septic-associated complications, such as immunosuppression, and vascular and cardiac damage. Therefore, circRNAs are set to be appealing for research in sepsis in the near future, and we envision a promising future in the diagnosis, prognosis, and theragnosis of this life-threatening condition.

## Figures and Tables

**Table 1:** Key circRNAs controlling the main underlying mechanisms in sepsis.

CircRNA	Mechanism	Role	Reference
mcircRASGEF1B	Inducible with LPS stimuli during microbial infection through TLR4	Protects cells against microbial infection by regulating the stability of mature ICAM-1 mRNAs	[138]
Circ-010231	Regulates different immune responses to virus	Plays an important role in host defense to virus by inducing competitive binding between host circRNAs and viral mRNAs	[126]
Circ_0005105	Interacts with the mRNA of pro-inflammatory cytokines	Induces a pro-inflammatory phenotype	[103]
Circ RNA-CER	Induced in chondrocytes by IL-1 and TNFα	Mediates an inflammatory response through interaction with IL-1 and TNFα	[136]
Circ_0028644	Regulates the expression of NF-κB	Modulates different pro-inflammatory phenotypes	[101]
Circ_4099	Modulates the expression of miR-616-5p	Closely related to inflammatory phenotypes through TNF- α	[100]
Circ_0003159	Regulates miR-223	Induces an inflammatory response due to increased expression of IL-6, IL-1β, and TNF-α	[111]
Circ_RSF1	Regulates the expression of inflammatory cytokines	Represses the interactions of miR-146a with RAC1 by eliminating its inhibitory effect on the RAC1 pathway	[115]
Circ_102685	Modulates the expression of miR-146a	Plays a role in endotoxin tolerance, immunosuppression, inflammatory response, and antiviral pathways	[116]
Circ_0005075	Regulates miR-23a-5p and miR-23b-5p	Suppresses the expression of miR-23b-5p in cancer and is related to immune response	[123,124,125]
Circ-PVT1	Interacts with the miR-125 family	Exhibits a possible correlation with sepsis severity, inflammation, and increased mortality	[117]
Circ-GLI2	Negatively regulates the expression of miR-125b-5p	Involved in inflammation and immune response pathways	[119]
Circ-MYLK and Circ_CTDP1	Regulates miR-29a-3p	Feasible predictive biomarker for assessing 28-day mortality of sepsis patients	[139,140]
Circ_HIAT1	Regulates miR-29a-3p and miR-29c-3p, and matrix metrix metalloproteinases MMP-9 and MMP-2	Increases miRNAs stability	[141]
Circ_NSD2	Regulates different processes through sponge miR-199b	Related to the low miR-199b-5p levels found in sepsis patients	[142]
Circ_0005785	Regulates miR-181a and miR-181b	Possible role in sepsis by promoting immunosuppression in late sepsis	[127]
Circ_0000096	Regulates the expression of miR-224 and miR-200a	Modulates the immune response through cyclin D1, CDK6, MMP-2, and MMP-9	[143]
Circ_001569	Modulates the expression of miR-145	Involved in the immune response of host to pathogens	[139,140]
Circ_HIPK3	Modulates the expression of miR-193a-3 and miR-124	Mediates a pro-inflammatory state by modeling the inflammatory response through sponge miR-124 (inhibitor of IL-6)	[144]
Circ_0003528, Circ_0007196 and Circ_0078738	Interacts with miR-192-5p	Related to the low levels found in sepsis patients	[145]
Circ RNA-9119	Modulates miR-26a	Increases the expression of PTGS2 by modulating the response of endothelium	[146]
Circ_TRIM33 and Circ_FOXO3	Modulates the expression of miR-191 and induces the expression of TET1	Induces proliferation, migration, and immune regulation	[147,148,149]
Circ RNA_007893	Regulates the expression of IL-6	Regulates the expression of IL-6, through sponging and endogenous miR-485-5p	[102]
Circ MAN2B2	Regulates the expression of S1000A8	Modulates immunosuppressive states	[128]
Circ RNA-MSR	Modulates miR-27	Induces pro-inflammatory phenotype	[121]
Circ_0012919	Increases the expression of DNMT1	Modulates immune response by reducing the expression of CD70 and CD11a in CD4^+^ T cells	[104,150]
